# CRISPR/Cas9—Advancing Orthopoxvirus Genome Editing for Vaccine and Vector Development

**DOI:** 10.3390/v10010050

**Published:** 2018-01-22

**Authors:** Arinze Okoli, Malachy I. Okeke, Morten Tryland, Ugo Moens

**Affiliations:** 1 Biosafety of Genome Editing Research Group, GenØk-Centre for Biosafety, Siva Innovation Centre, N-9294 Tromsø, Norway; malachy.okeke@uit.no (M.I.O.); morten.tryland@uit.no (M.T.); 2 Artic Infection Biology, Department of Artic and Marine Biology, The Artic University of Norway, N-9037 Tromsø, Norway; 3 Molecular Inflammation Research Group, Institute of Medical Biology, The Arctic University of Norway, N-9037 Tromsø, Norway; ugo.moens@uit.no

**Keywords:** CRISPR/Cas9, genome editing, modified Vaccinia virus Ankara, orthopoxvirus, recombination, risk assessment, site-specific, Vaccinia virus, vaccine, vector

## Abstract

The clustered regularly interspaced short palindromic repeat (CRISPR)/associated protein 9 (Cas9) technology is revolutionizing genome editing approaches. Its high efficiency, specificity, versatility, flexibility, simplicity and low cost have made the CRISPR/Cas9 system preferable to other guided site-specific nuclease-based systems such as TALENs (Transcription Activator-like Effector Nucleases) and ZFNs (Zinc Finger Nucleases) in genome editing of viruses. CRISPR/Cas9 is presently being applied in constructing viral mutants, preventing virus infections, eradicating proviral DNA, and inhibiting viral replication in infected cells. The successful adaptation of CRISPR/Cas9 to editing the genome of Vaccinia virus paves the way for its application in editing other vaccine/vector-relevant orthopoxvirus (OPXV) strains. Thus, CRISPR/Cas9 can be used to resolve some of the major hindrances to the development of OPXV-based recombinant vaccines and vectors, including sub-optimal immunogenicity; transgene and genome instability; reversion of attenuation; potential of spread of transgenes to wildtype strains and close contacts, which are important biosafety and risk assessment considerations. In this article, we review the published literature on the application of CRISPR/Cas9 in virus genome editing and discuss the potentials of CRISPR/Cas9 in advancing OPXV-based recombinant vaccines and vectors. We also discuss the application of CRISPR/Cas9 in combating viruses of clinical relevance, the limitations of CRISPR/Cas9 and the current strategies to overcome them.

## 1. Introduction: Orthopoxviruses in Vaccines and Vector Development

The genus Orthopoxvirus (OPXVs) (family Poxviridae, subfamily Chordopoxvirinae) contains, among others, Vaccinia virus (VACV)—the type species of the genus, *Variola virus* (VARV)—which caused smallpox, Monkey poxvirus (MPXV) and Cowpox virus (CPXV). Orthopoxviruses (OPXVs) are large (200 × 250 nm) brick shaped enveloped viral particles with a large genome (170–250 kb), which unlike most DNA viruses, replicate in the cytoplasm of the host cell. Whereas some OPXVs are host specific (e.g., VARV), others have broad host range and are also zoonotic (e.g., CPXV, VACV and MPXV).

Orthopoxviruses (OPXVs) are suitable viral vectors because they have large transgene capacity (up to 25 kilobasepairs (kbp) [1]); broad host range (including humans) [2,3]; stimulate long-term cellular and humoral immune responses to the vectored antigen(s) despite pre-existing vector-backbone immunity [1,4]; thermostable as a freeze-dried compound; easy to store, ship and use; and cost effective to manufacture. The first OPXV successfully used as a vector was Vaccinia virus (VACV). VACV was originally applied as a vaccine agent to eradicate smallpox. The vaccination programme (WHO 1966–1980), which involved hundreds of millions of people over a large geographical area and long period of time, generated a substantial pool of knowledge and experience on the effectiveness and side effects of VACV. Following the induction of protective immunity against hepatitis B virus infection by a recombinant VACV encoding hepatitis B surface antigen [5], VACV and replication-defective VACV variants (e.g., modified Vaccinia virus Ankara (MVA) and Vaccinia virus Copenhagen (NYVAC)) became attractive for the development of recombinant vaccines/vectors against a wide range of human and veterinary diseases [6,7]. Recombinant OPXVs encode antigens from one or several infectious agents, antigens relevant for cancer, and genes encoding specific immune stimulating factors such as cytokines/chemokines [8]. 

The broad host range of OPXVs may represent a potential risk of spill-over to non-target species, which is not always beneficial, but also allows to reach multiple target species, such as during the deployment of Raboral V-RG (RVG), a VACV-based recombinant Rabies vaccine that targets multiple wildlife reservoir species for rabies control and eradication programs in Europe and North America [9,10]. A major argument against the use of live OPXVs as vaccine vectors has been their potential of spontaneous recombination events with naturally occurring virus relatives, such as CPXV [11,12,13]. Single nucleotide polymorphisms (SNPs), insertions, deletions and genome rearrangements are commonly detected especially around the terminal inverted repeats, which affect the genome size and may be associated with altered infectivity and pathogenicity [14,15]. Although the replication-defective OPXV vectors are considered safer compared to replicative competent VACV, it is possible that a replicative-deficient and thus non-infectious virus may be recovered by the presence of a different poxvirus in the same cell; e.g., Shope fibroma virus can reactivate VACV DNA in infected and transfected cells [16,17]. These mechanisms are relevant when biosafety issues associated with open use or release of recombinant OPXVs are considered [18,19]; see also our recent review on biosafety considerations of MVA [20].

## 2. Classical Methods for Generating Orthopoxvirus Recombinants

### 2.1. Methods

**Figure 1:**
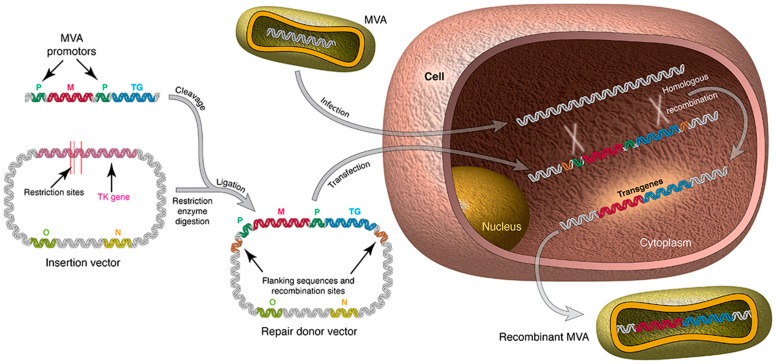
Construction of recombinant MVA vector by homologous recombination. A plasmid that contains the gene or transgene of interest is constructed and used to transfect an MVA-infected cell. TK^-^ Recombinant MVA vectors are produced by homologous recombination; TK: thymidine kinase gene; M: marker gene; TG: foreign gene; P: promoter; O: Origin of plasmid replication; N: Marker gene for plasmid selection.

Recombinant OPXVs can be constructed by homologous recombination between transfected heterologous DNA and replicating virus DNA [21], in vitro ligation [22], or by bacterial artificial chromosome (BAC) recombineering [23]. In homologous recombination, which is the popular classical method for modification of OPXVs, the gene(s) to be inserted is cloned into a plasmid transfer vector and is flanked by OPXV sequences that direct recombination to the desired locus (Figure 1). Transfection of the plasmid transfer vector into OPXV infected cells will result in homologous recombination between the replicating OPXV DNA and the plasmid vector, resulting in the insertion of the transgene into the OPXV genome (Figure 1). Commonly used insertion sites include the *thymidine kinase* (*TK*) gene, *haemagglutinin* (*HA*) gene, intergenic region between the *F12L* and *F13L* genes as well as naturally occurring deletions sites in the OPXV genome (especially with regard to MVA) [21]. Clonal isolation of recombinant viruses can be based on colour (fluorescent proteins or immunostaining) [21,24], antibiotic resistance [25,26], transient host range [27,28], plaque size [29,30] or complementation [21,31]. Several rounds of plaque purification are needed to obtain a pure clone of the desired recombinant virus as the current General Manufacturing Practice (cGMP) and guideline (e.g., the European Union Directive 2001/18/EC) for virus-vectored vaccines require that the scaled-up batch of the recombinant virus must be marker free and free of mutation in the expression cassette and flanking sequences.

Alternatively, in vitro ligation can be used to generate the transgene of interest with desired flanking regions by PCR, and this naked DNA sequence is transfected into cells infected with OPXV [32] or direct in vitro ligation to create chimeric OPXV DNA. In this method, the OPXV genome is cleaved at unique restriction endonuclease sites and a transgene expression cassette can be directly ligated to produce a recombinant DNA. Since OPXV DNA is not infectious, viruses with the modified recombinant DNA are recovered by transfecting the chimeric DNA molecules into cells infected with a helper poxvirus [22]. BAC recombineering is another approach for generating chimeric poxviruses. In this method, the entire OPXV genome is cloned as a BAC and the major steps involved include; (i) generation of pre-BAC clones by insertion of mini-F plasmid shuttle vector into the OPXV genome; (ii) isolation of BAC clones from pre-BAC OPXV DNA; (iii) amplification of the BAC clones (BAC miniprep); (iv) gene editing by Red recombineering in *E. coli* and (v) rescue of the genomic BAC clone with a helper fowlpox virus [33,34]. For clinical applications, BAC clones of OPXV vectors must be free of marker genes, mini-F plasmid and any other bacterial sequence. These unwanted sequences are usually removed by *en passant* mutagenesis [35], Cre/LoxP or FLP/FRT (Flippase/Flippase Recognition Target) recombination systems [36,37].

### 2.2. Limitations

Generally, these systems require tedious laborious multi-steps with low efficiency among other limitations. Generating candidate recombinant vaccines by homologous recombination is limited by; (i) low recombination efficiency (<3%); (ii) time consuming processes such as generating the plasmid with the transgene and plaque purification of the recombinant virus; (iii) transgene instability upon virus expansion; (iv) requirement for 200–500 bp of flanking DNA sequence (which favour recombination into off-target sites); and (vi) lack of multiple editing of several genes in parallel [21,22,38,39,40,41]. Although in vitro ligation obviates the need for cloning in bacteria, the introduction of marker genes to improve selection efficiency can be labour intensive and technically demanding. In addition, this method cannot be used to edit every gene in the OPXV genome due to the lack of unique restriction endonuclease sites across the genome [40]. At present, no OPXV-vectored vaccine intended for clinical trials or marketing authorization application has been generated by direct in vitro ligation [22]. Unlike homologous recombination, BAC recombineering requires less than 50 bp of flanking arms, generates marker free recombinants without time consuming plaque purification, and allows editing of multiple genes in parallel as well as the isolation of fitness-disadvantaged mutants [33]. However, BAC recombineering poses the risk of insertion of bacterial sequences and transposons into BAC clones as well as the potential risk of recombination between the BAC clone genome and the helper poxvirus during virus reconstitution [34]. Currently, no poxvirus-vectored vaccine generated by BAC recombineering is in clinical development although the method has been used to demonstrate that the six major deletions in the genome of the MVA vector are not sufficient for its host range defect in most mammalian cells [42]. Thus, for convenience, effectiveness, cost and time, a more efficient and straightforward approach to editing OPXV genomes for generating recombinants would be beneficial to vaccine and vector development.

## 3. CRISPR/Cas9—A New Addition to Modern Genome Editing Toolbox

The CRISPR/Cas9 (Clustered Regularly Interspaced Short Palindromic Repeats (CRISPR)/associated protein 9) is one of the latest introductions to the tools of modern genome editing. Derived from the *Streptococcus pyogenes* Type II CRISPR/Cas system [43,44] where the gRNA (guide RNA)-guided *cas* gene targets and breaks DNA at specific sequences [45,46], CRISPR/Cas9 has been adapted to editing genomes of virtually any organism [43,44,47]. In bacteria and archae where it plays an important role in the adaptive immune defence process, CRISPR/Cas activity is generally characterized by (1) adaptation—which leads to insertion of new spacers in the CRISPR locus; (2) expression—which primes the system for action by expressing the *cas* gene and transcribing the CRISPR into, first a precursor CRISPR/RNA (crRNA), and then a mature crRNA; (3) interference—during which the target nucleic acid is recognized by its PAM (a conserved dinucleotide-containing Protospacer Adjacent Motif sequence upstream of the crRNA binding region) and nicked by the combined action of crRNA, a transactivating CRISPR RNA (tracrRNA) and Cas proteins [48]. In CRISPR/Cas9, crRNA and tracrRNA are fused to form sgRNA (single guide RNA) [45,46], which directs the Cas9 to the specific DNA sequence to be cut (Figure 1).

**Figure 2:**
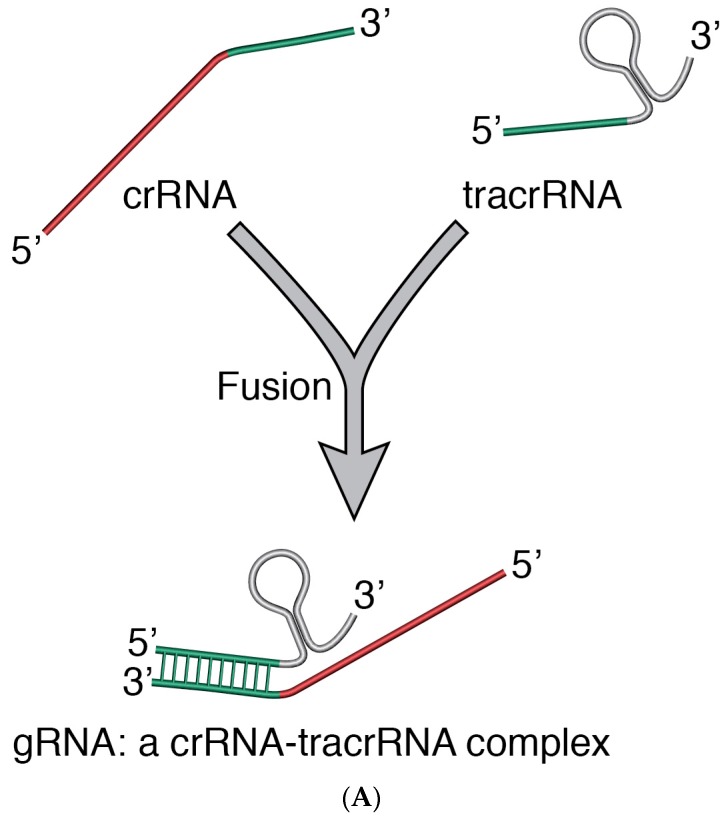

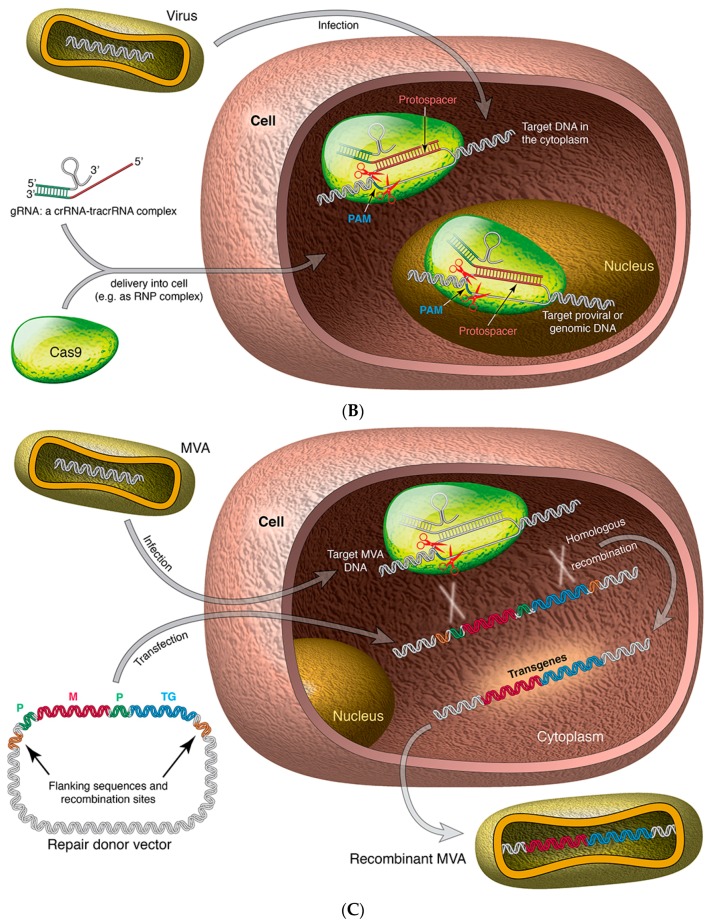
(**A**) crRNA and tracrRNA are fused to form the sgRNA; (**B**) sgRNA interacts with Cas9 and with a section (a short homologous sequence of about 20 nt –protospacer) on the target DNA (e.g., a virus, provirus or genomic DNA), thus directing the Cas9 to a specific site on a target DNA. The Cas9 nuclease activity results in a double stranded cut (indicated with the scissors) in the target DNA; the cut stimulates the cell’s DNA repair mechanism. RNP Complex: Cas9/gRNA Ribonucleoprotein. (**C**) In the presence of a DNA template with flanking sequences homologous to the cut regions of a target DNA, the Homology Directed Repair (HDR) mechanism can be activated and be exploited to generate a recombinant virus, e.g., recombinant MVA. M: marker gene; TG: foreign gene; and P: promoter.

The repair of the specific single double-stranded break made by CRISPR/Cas9 in a targeted region of the DNA by the preferred Non-Homologous End Joining (NHEJ) is error prone. Thus, during repair by this pathway, substitutions, insertions and deletions (indels) often occur leading to frameshift or premature stop codon that can inactivate the gene [49,50]. The less preferred Homology Directed Repair (HDR) can be induced in the presence of a homologous gene template (Figure 2C), which is precisely incorporated into the cut region via homologous recombination [49,50]. Either or both pathways can be manipulated for genome editing by the CRISPR/Cas9 system—NHEJ for gene knock-out, and HDR for gene knock-in. CRISPR/Cas9 technology has been successfully used to introduce changes in the genomes of viruses [51], bacteria [52], yeasts [53], plants [54], and animals [55,56,57]. This technology is now widely explored as a therapeutic strategy against infections [58] (Table 1 and Table 2, Section 2.1 and Section 2.2), various non-malignant and malignant diseases [59,60,61,62,63], and in vaccine development and gene therapy [64,65]. In cancer treatment, clinical trials of CRISPR/Cas9-based therapy have been initiated [63], and several preclinical studies involving CRISPR/Cas9-mediated correction of human genetic diseases are underway [66].

## 4. Applications of CRISPR/Cas9 in Genome Editing of OPXVs and Other Viruses of Clinical Relevance

### 4.1. Targeted Editing of Virus Genomes

Viruses depend on host factors for their replication, thus, it was more challenging, compared to self-reproducing organisms, to adapt modern genome editing tools in editing virus genomes [80]. The success being recorded with CRISPR/Cas9 in generating virus mutants, inactivating viral replications and clearing viruses from infected cells (Table 1 and Table 2) can be attributed to the simplicity, flexibility, robustness and low cost of the technique (See Section 6). In VACV, CRISPR/Cas9 has been used to generate mutants [64] that can be employed as vaccine vectors against infectious diseases, and vectors for cancer treatment and gene therapy. The *TK* gene—whose deletion mutants are restricted to replication in cancer cells, and the *NIL* and *A46R* genes that play important roles in VACV virulence and host immune response have been edited using CRISPR/Cas9 [65], showing the ability of the system to edit VACV genome. In addition, in silico analysis revealed that virtually every gene in the VACV genome can be targeted by CRISPR/Cas9:sgRNA-directed site specific mutation, and multiple target sites for efficient HDR mediated homologous recombination have been identified in VACV genome [64]. However, CRISPR/Cas9 indels efficiency of specific genes of the cytoplasmic replicating VACV can be low, as exemplified for *N1L* and *A46R* where the efficiency of specific mutations was ~10% [64], unlike the higher efficiency (50%) in Adv-EGFP (Adenovirus-Enhanced green fluorescent protein) when EGFP was targeted, and (47.5%) in Herpes Simplex virus type-1 (HSV-1) when the *TK* gene was targeted [79]. Both Adv and HSV-1 replicate in the nucleus where the NHEJ mechanism is more efficient. Nonetheless, high efficiencies (62.5% and 85% respectively) of HDR-mediated CRISPR/Cas9 editing at the same *N1L* and *A46R* gene loci in the presence of a template for recombination (the tumour-associated antigen, TRP2, flanked by homologous sequences targeting both sides of *NIL* and *A46R*) were achieved using a plasmid encoding Cas9 without nuclear localization signal [64]. 

Direct targeting and precise inactivation of proviral genomic regions of the human immunodeficiency virus (HIV-1), Epstein-Barr virus (EBV), JC polyomavirus (JCPyV), herpes simplex virus type 1 (HSV-1), hepatitis B virus (HBV), and human papilloma virus (HPV-16) in infected cells by CRISPR/Cas9 have been achieved with varying degree of success (Table 1). For example, in HIV-1, genes relevant for the virus infection, replication, and escape have been successfully disrupted (Table 1). In a particular study, the entire proviral genome spanning 5′-3′ Long Terminal Repeats was precisely removed from latently infected human CD4^+^ T cells [72], and further infections were prevented by persistent co-expression of Cas9 and sgRNA in the HIV-1-eradicated cells [111]. Disruption of HIV-1 replication and inhibition of viral infection at early phase has also been achieved by CRISPR/Cas9:sgRNA system [74,75]. Similarly, in HSV-1 and HBV, precise disruption or removal of genes that are relevant for virus replication [81,88,89], recombination [82], or reduce expression of specific genes leading to virus clearance [90] have been achieved (Table 1); see also [140] for a review on application of CRISPR/Cas9 against human viruses. Targeting multiple genomic locations achieved better disruption of virus replication and prevented development of mutants that are resistant to the sgRNA in HIV-1 [70,73,111], HSV-1 [80,81], HBV [88,89,90], and EBV [67,68,81].

### 4.2. Virus-Host Interaction

Another strategy to interfere with viral infection is CRISP/Cas9-mediated targeting of host cell factors crucial for the viral life cycle. CRISPR/Cas9 mediated disruption of genes expressing receptors or co-receptors required for viral infection can protect host cells against infection. This has been demonstrated for HIV-1 co-receptors CCR5 [107,114,115,141,142] and CXCR4 [117], the poliovirus receptor [128], the HCV receptor molecules CD81, occluding (OCLN) and claudin-1 (CLDN1) [104,143], and the AXL receptor for Zika virus in HeLa cell infection [131]. However, ablation of AXL in human neural progenitor cells had no effect on Zika virus entry [129]. Hence, the use of AXL as a receptor by Zika virus may be cell-specific. 

CRISPR/Cas9 genomic editing can also be used to identify cellular proteins acting as anti-viral defense molecules. Disruption of the genes encoding IRF3, STAT1, IPS1 or STING showed that these proteins are important in anti-viral response to the alphaviruses Chikungunya virus, Venezuelan equine encephalitis virus, and Sindbis virus infection [133]. STAT1 and STAT2 are required for inhibition of HCV replication by IFN-λ, while only STAT2 is involved in IFN-α induced inhibition of HCV replication [102]. CRISPR/Cas9-mediated knockout of the TSPO gene (encoding mitochondrial translocator protein) or MAN1B1 gene (encoding endoplasmic reticulum Class 1α mannosidase) in HEK293T cells demonstrated that these proteins are implicated in degradation of HIV-1 Env, resulting in inhibition of HIC-1 replication [119,120]. 

Alternatively, CRISPR/Cas9 can be used to prevent that the virus evades inflammation and the immune response. This strategy has been successfully applied to enhance the immune response against respiratory syncytial virus (RSV). RSV can infect mesenchymal stem cells (MSCs), which are known to regulate immune response via immune regulatory factors, including cytokines, interferons, inducible nitric oxidase, and indoleamine-2,3-dioxygenase 1 (IDO-1). RSV infection of MSCs resulted in ~70-fold increase in IDO-1 protein levels [136]. Conditioned medium from RSV infected MSCs gave significantly lower proliferation of peripheral blood mononuclear cells (PBMCs) compared to conditioned medium from mock infected cells. The authors showed that CRISPR/Cas9-mediated knockout of the IDO1 gene in MSCs cells prevented the anti-proliferative effects of conditioned medium from RSV-infected MSCs, and concluded that that RSV-induced expression of IDO might diminish the protective immunity against RSV infection. 

Genomic editing is an elegant technique to identify host cell proteins that are involved in viral replication. The CRISPR/Cas9 method allowed the identification of a luminal domain such as LAP1 (LULL1) as a crucial host protein in assembly and packaging of HSV-1 [105], while cellular protein kinase R blocks HCMV replication [137]. HCV replication is inhibited by the ubiquitin-like protein ISG15 [103], while knockout of cellular DNA polymerase K prevents conversion of relaxed circular HBV DNA into covalently closed circular DNA and subsequent HBV replication [98]. BST-2 or tetherin was shown to prevent the budding of HIV-2 [121]. HIV-1 production is reduced and viral export is impaired in cells where the endosomal sorting complex for transport II protein EAP45 has been ablated [118], whereas knockout of the *SAMHD1* gene, encoding the deoxynucleoside triphosphate triphosphohydrolase SAM domain- and HD domain containing protein 1 (a), increased HIV-1 infection [110].

Genomic editing has also identified several cellular proteins used by members of the *Flaviviridae* family. West Nile virus, Dengue virus, Zika virus, Yellow fever virus, Japanese encephalitis virus, and HCV replication all depended on genes whose products are associated with endoplasmic functions such as translocation, protein cleavage, and N-linked glycosylation (e.g., OSTC, STT3A, SEC61B, SEC63, SPCS1, SPCS3, translocon-associated protein complex proteins SSR1, SSR2 and SSR3), as well as in endocytosis (RAB5C, RABGEF, WDR7, ZFYVE20), posttranslational modification (NDST1, SST3A, EXT1 and EXT3), and in transmembrane processing and maturation (EMC1-10, SSR2, and SSR3) [131,132,143]. Moreover, HCV replication depended on RNA binding proteins (e.g., ELAVL1) and enzymes involved in metabolism such as riboflavin kinase and flavin adenine dinucleotide synthetase 1 [143]. Translation of Sindbis virus subgenomic mRNA did not require eIF2A and eIF2D [139]. The calnexin and calrectulin proteins, which are part of the reticulum chaperone system, are required for efficient Ebolavirus glyprotein production [127]. Genomic editing of >10,000 genes in HeLa cells by CRISPR/Cas9 identified the ST3GAL1 (ST3 β-galactoside α-2,3-sialyltransferase 1), STGAL 4, *COG1* and *COG5* (encoding component of oligomeric Golgi complex 1, respectively complex 5), and MGAT5 (mannosyl (α-1,6-) glycoprotein β-1,6-*N*-acetyl-glucosaminyltransferase) as essential host genes for enterovirus replication [128]. The cellular proteins SAMD9 and WDR6 form host restriction factor that prevent VACV replication in human cells [144]. CRISPR/Cas9-mediated mutation of the gene for glucose-regulated protein 78 (GRP78), an endoplasmic reticulum chaperone, enhanced hepatitis A virus replication [93]. 

### 4.3. Cellular Genes and Viral Induced Tumorigenesis and Pathogenicity

Finally, host cell genome editing unveiled cellular genes involved in the virulent properties of viruses. CRISPR/Cas9-mediated ablation of p53 and PTEN accelerated liver tumorigenesis in HBV transgenic mice [94], while HBV-mediated upregulation of cellular microRNA miR-3188 promoted cell proliferation, cell growth, migration, and invasion of HCC cells [135]. Moreover, CRISPR/Cas9-based studies showed that the HBV protein HBX stimulates proliferation and cell mobility, and inhibits apoptosis of the hepatocellular carcinoma HuH-7 cells via the small GTPase CDC42 [97]. CRISPR/Cas9 editing showed that the cellular protein CD63 is involved in exosomal transmission of the Epstein-Barr virus (EBV) latent membrane protein 1 (LMP1) oncoprotein [123] and identified 57 cellular genes in EBV-dependent Burkitt’s lymphoma and 87 genes in EBV-infected lymphoblastoid essential for cell growth and survival [122]. These genes encode among others, proteins involved in signal transduction, tumour suppressors, cell cycle control and cell survival. CRISPR/Cas9-mediated knockout of the NAD^+^-dependent protein deacetylase SIRT1, a potential oncoprotein, suppressed proliferation and colony formation in soft agar of KSHV-transformed cells. These findings suggest that SIRT1 contributes to KSHV-induced tumorigenesis [134]. The genes *EMC2*, *EMC3*, *SEL1L*, *DERL2*, *UBE2G2*, *UBE2J1*, and *SYVN1* whose products belonged to the endoplasmic reticulum-associated protein degradation pathway protected against West Nile virus- and Saint Louis encephalitis virus-induced cell death. However, knockout of these genes did not block viral replication [145].

## 5. CRISPR/Cas9: A Veritable Tool for Advancement of OPXV-Based Vaccines and Vectors?

Several OPXV-based recombinant vaccines and vectors are currently at different stages of clinical trials. Many of the vaccines and vectors are based on VACV, MVA, NYVAC, Raccoon poxvirus, and modified Vaccinia Tian Tian (MVTT) OPXV strains, and they target malignancies (e.g., prostate, skin, colorectal, breast and ovarian cancer) [146,147,148,149]) and infectious diseases (e.g., AIDS, malaria, ebola, tuberculosis, hepatitis, influenza) (Supplementary Table S1). In many parts of Europe, Canada and USA, a VACV recombinant vaccine–Raboral V-RG was deployed to eradicate Rabies virus from the wild fox population [9,10], and several OPXV-based vectored vaccines have been used in preventing animal diseases [150]. Despite these advances, none of the OPXV-based recombinant vectors or vaccines has been licensed for human use. The main drawbacks include low predictability of attenuation; sub-optimal immunogenicity; transgene instability; potential for reversion of attenuation or to wild-type strain; potential of transmission to non-target hosts; and exchange of genetic materials with viral strains in the environment [20], which are some of the considerations for efficacy, patient’s safety and environmental safety during evaluation of genetically modified vaccines and vectors for approval [18,19,20]. Can the CRISPR/Cas9 system facilitate the development of OPXV-based recombinant vaccines with a superior level of immunogenicity, limited potential to spread to non-target host, relatively stable against reversion of attenuation, high predictable level of attenuation not offered by the classical methods? 

### 5.1. Genome and Transgene Stability

Apart from arming the recombinants with the transgenes against the targeted disease, several strategies to increase the efficacy, e.g., immunogenicity, of OPXV-based recombinant vaccines and vectors include insertion of immunomodulatory and co-stimulatory genes, and gene deletion both to attenuate and increase immune induction. In MVA the immunomodulatory genes targeted for deletion include *146R* [151], *153L* [152], *157L* [153], *159R* [153], *183R* [154], *184R* [154], *O19L* [155], and *050L* [156]. In many cases, several genes are deleted in parallel. This strategy has been used to improve antigen presentation, priming of immune cells and subsequent synthesis of immune effectors and host response to transgenes [152,153,154,155]. For oncolytic VACV and MVA recombinants, knock-out (or replacement with intended transgene) of the *TK* gene to restrict virus replication to tumour cells is an additional strategy to ensure safety. A genome editing system such as CRISPR/Cas9:sgRNA that requires few virus multiplication cycles will reduce selection pressure on the vectored vaccine. For example, the *TK* gene was replaced (with the red fluorescent protein (RFP)) at a greater than 90% rate in VACV [65]; further, several sgRNA target sites have been mapped on VACV genome enabling targeting of multiple genes in parallel. The technology has been applied to effectively develop an efficient anti-H5N1 polyvalent duck vaccine within 3 weeks [157]. High selection pressure often results when recombinants must be passaged through multiple cycles, especially in knock-in of several antigens for polyvalent vaccines/vectors. With the current classical methods, it can take up to 10 rounds (lasting 4–6 weeks) of plaque purification (with low success rate) of obtaining the desired recombinant [158]; but with CRISPR/Cas9, desired VACV recombinants were obtained in 3 rounds of purification [64]. Indeed, the CRISPR/Cas9 in combination with Cre/Lox system has been used to develop a stable anti-pseudorabies virus (PRV) vaccine of pig [159]. Recombinant PRV with double gene deletion was obtained in a single round of plaque purification (instead of 10 rounds of plaque purification by traditional strategy), which enhanced both the efficacy and stability of the recombinant vaccine [159]. Transgene instability (mutation in the insert) and/or genome instability (mutation outside the insert) in recombinants can result from high selection pressure due to multiple passage cycles. Instability in rMVAs have been reported [41,160,161]; in one of the reports [160] the transgene was completely lost. Transgene/genome instability can compromise the efficacy of the vaccine; and loss transgene(s) will impede post-release monitoring or monitoring of escaped recombinants given that the transgene(s) is the tag for tracking the recombinants. 

### 5.2. Predictability of Attenuation and Host Range Restriction

Apart from history of safe use, the good safety profile of MVA is predicated on the virus’s host range restriction—being unable to produce progeny viruses in human cells and most mammalian cell lines. However, the molecular basis for MVA’s host restriction has yet to be determined because the specific gene deletions and mutations that are responsible for the lack of full replication in most cells have not been identified [42], although the deletions and mutations responsible for the virus overall attenuation are known [162]. For example, the finding, using BAC recombineering, that the six major VACV gene deletions in MVA were not sufficient for the latter’s restricted host range indicates the existence of other culprits, but the limitations of BAC and the other classical methods have been a hindrance in elucidating these [42]. In addition, the exact roles of the several mutations across the genome of MVA have not been deciphered. At the moment, production of viral progenies in human vaccinees and subsequent spread to non-target hosts cannot be completely ruled out because only a limited range of human and mammalian cell lines have been tested for full virus multiplication; moreover, in some human cell-lines, e.g., HeLa and HEK293, semi-productive infections have been reported [163,164]. The advantages of CRISPR/Cas9 system can be exploited to elucidate the molecular basis of MVA attenuation and host range restriction. This information will help to develop measures to avoid or reduce spread of rMVAs and transgenes to non-target hosts and the environment.

### 5.3. Elucidation of Factors that Influence rMVA Vaccines and Vectors

In addition to lack of knowledge of the specific mutations that determine MVA host range restriction, knowledge gaps exist in the study of virus and host factors that influence rMVA vaccines/vectors. Research is currently underway to establish how transgene stability is affected by expression levels of transgene, timing of transgene expression, transgene insertion site, and sequence/structure of the transgene/flanking region [161,165,166]. Also, more research is required to elucidate how rMVAs are influenced by promoter choice and promoter spacer length [41,167], and host cell used for recombinant virus amplification [11]. Given the simplicity with which recombinant VACV was generated by the CRISPR/Cas9 system, an exponential increase of its application in basic research targeting the highlighted issues in generating recombinant OPXVs is expected. Filling these knowledge gaps will advance the design of rMVA vaccines and vectors; it will also facilitate the risk assessment of such products.

## 6. CRISPR/Cas9 Versus Other Modern Genome Editing Tools

Currently, CRISPR/Cas9 technology is being applied more extensively than TALENs (Transcription Activator-Like Effector Nucleases), ZFN (Zinc Finger Nucleases), ODM (Oligonucleotide Directed Mutagenesis), Cre/Lox and FLP-FRT recombination systems. TALENS and ZFN were previously considered the best programmable and precise techniques for genome editing [168]. Like CRISPR/Cas9, both TALENs and ZFN are chimeras of sequence-specific DNA-binding guides that are linked to a non-specific DNA cleavage nuclease. However, unlike Cas9 which is an RNA-guided nuclease, Fok1 in TALENs and ZFN are guided by a protein. TALENs are fusions between the FokI DNA cleavage domain and DNA-binding domains derived from TALE proteins, while ZFNs are fusions between the FokI DNA cleavage domain and zinc-finger proteins. Both TALENs and CRISPR/Cas9 are based on bacteria secretion systems (TALENs on the Genus *Xanthomonas* bacteria Type III secretion system [169]; CRISPR/Cas9 on *Streptococcus pyogenes* Type II secretion system [43,44]). ZFN and TALENs both share the similarity of the use of Fok1 restriction endonuclease.

The genomes of HIV-1, HPVs, HSV-2 and HBV have also been edited using TALENs and ZFN [170,171,172,173,174], but to a lesser extent compared to CRISPR/Cas9. All three genome editing tools are associated with off-target effects, sub-optimal efficiency in non-bacterial systems and generation of escape viruses, however, CRISPR/Cas9 achieves a much higher efficiency (up to 85% for HDR mediated CRISPR/Cas9 knock-in in VACV [64]) than TALENs and ZFN. In addition, CRISPR/Cas9 is relatively easy to design and construct requiring only the fusion of a 20-nucleotide genomic target site into the overall sgRNA. Further, algorithms are available to predict putative off-target sequences of a sgRNA, and the Cas9 nuclease is re-usable. On the other hand, custom design and synthesis of TALENS and ZFN, which are based on the rearrangement of their modular DNA-binding domains, require labourious cloning techniques and rigorous testing [175,176]. Further, the cost of the CRISPR/Cas9 system is much lower—approximate cost required to generate a single, gene specific candidate CRISPR/Cas9 reagent is <100 USD compared to circa 1000 USD for TALENS and 5–10,000 USD for ZFN [177]. These advantages have made the CRISPR/Cas9 system more robust and applicable to overcoming the challenges related to virus genome editing. The superiority of CRISPR/Cas9 notwithstanding, researchers are combining some properties of TALENS and ZFN with CRISPR/Cas9 to achieve an improved system. For example, Cas9 has been fused to FokI (fCas9) to achieve reduced off-target effect of the CRISPR system [178].

## 7. Limitations of CRISPR/Cas9 in Virus Genome Editing

Sub-optimal efficiency in eukaryotic cells and viruses: high efficiency (up to 100%) of CRISPR/Cas system has been obtained in bacteria in which the system is naturally expressed [179], but in eukaryotic cells, e.g., human cell lines, efficiency of the *Streptococcus*-derived CRISPR/Cas9 varies between 2% and 38% [180,181], although studies with cells and living organisms have demonstrated up to 100% efficiency with improved CRISPR/Cas9 systems [49,182,183,184,185]. Beside not being a natural system in eukaryotes, CRISPR/Cas9 will be expected to be less efficient in editing the double alleles of a densely packed eukaryotic chromosome compared to the simpler haploid bacterial chromosome, but various strategies are being employed to improve efficiencies in non-bacterial systems. For viruses, the number of genome copies varies in infected cells—fewer genome copies will be present early in the infection cycle than later when viral genome replication has occurred. Virus replication site and multiplicity of infection (MOI) are other factors that have been reported to influence the efficiency of CRISPR/Cas9 in editing of virus genomes [64,65]. For example, and as stated in Section 4, the efficiency of NEHJ-mediated CRISPR/Cas9-induced specific indels in VACV was less than 10% [64], although for the HDR mediated homologous recombination at the same gene loci of *N1L* and *A46R*, the efficiencies were 60% and 94% respectively [64,65]. However, in HSV-1 the CRISPR/Cas9 indel efficiency at the *gE* gene locus was as high as 50%; and 47.5% in a recombinant adenovirus (Adv-EGFP) when enhanced green fluorescent protein (EGFP) was used as the target gene to be edited by Cas9 [79]. The lower genome editing efficiency observed in VACV was attributed to the site of replication being in the cytoplasm where the efficiency of NEHJ DNA repair mechanism is low compared to the nucleus. In Adv, genome editing peaked between 24 and 36 h post transfection [79], which are time points when viral genome replication had occurred with attendant high viral copies. High virus density—MOI of 1–10 was found optimal for Adv and VACV [64,79]. 

Furthermore, sgRNA concentration and sensitivity [79]; number of genes to be edited e.g., knock-in of single genes (e.g., 62.5% efficiency of *N1L* and 85% efficiency of *A46R*) was more efficient than the simultaneous knock-in of both genes (60% efficiency) [64]. Efforts, such as inhibiting the error prone NHEJ while giving a competitive advantage to HDR, are underway to improve the efficiency of the CRISPR/Cas9. For example, introduction of NHEJ inhibitor, SCR7, to the CRSPR/Cas9 system greatly increased its efficiency in gene knock-in editing of HSV-1 [80]. Also modifications in the current sgRNA structure such that it is closer to the structure of the bacterial tracrRNA has been shown to improve the efficiency of CRISPR/Cas9 knock-out genome editing [186].

Target specificity and off-target effect: the sequence specificity of the widely used Cas9 from *Streptococcus pyogenes* Cas9 permits up to four nucleotide base mismatches between the sgRNA and complementary sequence of the target nucleic acid, thus resulting in non-specific binding which often leads to cleavage of non-target regions of the genome [43,187,188]. Mismatch tolerance has been reported to generally depend on position of mismatched nucleotide in the sgRNA relative to the PAM sequence [189], concentration of sgRNA:Cas9 complexes, sgRNA length and activity [188]. Determinant of sgRNA: Cas9 specificity and thus off-target effect of a CRISPR/Cas9 system include sgRNA sequence construct, Cas9: sgRNA abundance, length and composition of PAM, nature of seed region, i.e., PAM-proximal 10–12 bases, accessibility and abundance of seed match genomic site and sgRNA scaffold; for a comprehensive review on determinants of Cas9:sgRNA specificity see [190]. For example, Bi et al. showed through sequence alignment that their sgRNA constructs for modifications of Adv and HSV-1 had different off-targets: 76 sgRNA-175 off-target sites are present in the human genome in which 19 are located in the exons of protein-coding genes [79]. The off-target sites for sgRNA-174, sgRNA-173, and sgRNA-206 that are present in the human genome are 123, 34, and 8, respectively [79]. However, the genome of viruses being smaller (most viruses have a genome between 3000–200,000 nucleotides compared to the 3 × 10^9^ in the human genome), fewer off-target effects can be expected. For example, when the sgRNA-175 sequence was aligned against the Adv and HSV-1 genomes, no significant homologous sequence was found [79]. Similar observation of no potential off-target region was obtained when the sgRNAs used in editing the VACV were aligned to the virus genome [64,65]. 

Several methods exist to detect off-target effects of the CRISPR/Cas9 technology (for a review see [191]). T7E1 cleavage assay, sequencing PCR-amplified potential off-target sites, whole genome sequencing (WGS) or exome sequencing are the most commonly used techniques. While WGS and exosome sequencing provides most sequencing data, these methods will identify variations throughout the complete or coding genome. Therefore, algorithms (e.g., CRISPR Design web server (http://crispr.mit.edu) that predict potential off-target sites should be used to authenticate whether de novo mutations in genomes are caused by CRISPR/Cas9-mediated genome editing events. Moreover, the mutation frequency may also indicate whether the mutations are the result of CRISPR/Cas9 or occurred spontaneously because the estimated spontaneous mutation frequency of the human genome is around 1.5 × 10^−9^ [192].

Various strategies are being devised to increase specificity and minimize CRISPR/Cas9 off-target effects. One strategy is to generate Cas9 nickase mutants, which required two sgRNAs on opposite strands flanking the target site for its double strand break activity [180,193,194]. A Cas9 nickase fused to cytidine deaminase has also been used to achieve site-specific single-base mutations in multiple gene loci [57]. Another strategy was to fuse a catalytically inactive Cas9 and the FokI endonuclease (fCas9) to produce an RNA-guided active FokI-dead-Cas9 nuclease [178,195,196]. Inhibition of Cas9 by anti-CRISPR protein AcrIIA4 has also been reported [197]. Truncation of sgRNAs such that they bear shortened regions of target site complementarity has also been used to reduce the off-target effect of CRISPR/Cas9 system [193,195,198]. Further, algorithms are being used to predict off-target sites in the viral genomes for a specific sgRNA [199].

Development of resistant escape virus variants: viruses have been reported to develop resistance to CRISPR/Cas9 or acquire revertant phenotypes over multiple infection cycles. Wang et al. [76] reported HIV-1 resistance to CRISPR/Cas9 in a viral evolution experiment using CD4^+^ T cells expressing Cas9/sgRNA that targets the HIV-1 genome. The group showed viral escape from Cas9/sgRNA on the basis of Cas9-induced indels in the targeted viral sequence [76]. The indels were not deleterious for viral replication, but were refractory to recognition by the same sgRNA in a different infection cycle as a result of changing the sequences of target DNA. Sites of resistance in HIV-1 induced by indels are common in the Cas9 cleavage sites [70,73,76,111]. Indels are more common in coding regions than non-coding regions; in the experiment by Yoder and co-workers, indels at targeted non-coding regions were single base-pairs, but were 3 base-pairs in coding regions [73].

Similarly, in human cytomegalovirus (HCMV) and HSV-1 where essential genes were targeted for editing by CRISPR/Cas9, virus variants that harbour mutations but still express functional proteins were detected [81]. This type of escape variants were able to bypass CRISPR/Cas9 editing by the same CRISPR/Cas9:sgRNA in subsequent passages [81]. However, when multiple essential genes were targeted using several sgRNAs, development of escape variants was prohibited [81]. Combinatorial CRISPR/Cas9 gene editing approach has also been used to halt development of escape variants [70]. Targeting sgRNAs to DNA sequences that are transcribed in codons for essential amino acids in the gene product could also help in prohibiting development of resistant variants, because any substitutions of these crucial amino acids will render the resultant proteins non-functional. Further, development of new Cas9 to cleave at sites outside the target has also been proposed as a strategy to inactivate resistant variants [200,201].

## 8. Conclusions and Future Prospects

The CRISPR/Cas9 system is revolutionizing genome editing approaches. Compared to other target-guided nuclease-based methods, the robustness, effectiveness, low cost and simplicity of CRISPR/Cas9 has made it easily adaptable to editing the genomes of almost any organism including viruses. The technique has been used to customize modifications in several viruses with the aim of generating recombinant mutants, inhibition of virus replication, excision of provirus genes or mutations in host cell receptors to prevent virus infection. The successful application of CRISPR/Cas9 to generate mutant VACV recombinants paves the way for its application in genome editing of other vaccine/vector-relevant OPXV strains. The technique can thus be applied in tackling some of the hindrances to approval of OPXV-based recombinant vaccines and vectors, which include sub-optimal immunogenicity, non-predictability of attenuation, reversion of attenuation, transgene/genome instability, potential of spread of transgenes to wildtype strains and/or close human and animal contacts of vaccinees or patients undergoing oncolytic therapies.

However, the full potential of CRISPR/Cas9 will be realised when several of the limitations of the technique including off-target mutations, escape virus variants and sub-optimal efficiency are overcome. Several improved CRISPR/Cas9 systems are already being developed, e.g., the Cas9 nickase variants which improves specificity by requiring two sgRNAs on opposite strands flanking the target site; the Cas9 nickase-cytidine deaminase fusion, which is used to achieve site-specific single-base mutation without requiring double strand breaks; the Fok1-dead-Cas9 nuclease -a catalytically inactive Cas9 employed to minimize the endonuclease activity of Cas9; and inhibition of Cas9 by AcrIIA4 to reduce off-target activity of Cas9. CRISPR/Cas9 can also be coupled to synthetic biology techniques, thus, enabling genome manipulation to the extent which the classical molecular biology techniques cannot, thus opening new frontiers in vaccine and vector development. 

Further, the full potential of the CRISPR/Cas9 system will be harnessed for vaccine and vector development if, from the early stage, research in the area of safety, including biosafety related to the use of the technique is also taken into consideration. Presently, the focus of research on CRISPR/Cas9 is predominantly on improving specificity and efficiency, and limiting off- and on-target effects of the system. Research is also required on the biosafety implications of off- and on-target effects of the CRISPR/Cas9 mutations, in particular, characterizing the outcomes of such unintended effects by coupling them to phenotypic changes in the virus and host. The results of such research will contribute to advancing the use of the technique in advancing OPXV-based vaccine/vector development. It will also help in the on-going debate in the European Union and other regions of the world on how to regulate products of CRISPR/Cas9.

## Figures and Tables

**Table 1:** Overview of CRISPR/Cas9 applications in genome editing of orthopoxvirus and other human viruses of clinical relevance.

Virus	Target in Virus Genome	Description	Reference
Vaccinia virus	*N1L*, *A46R*	Dual deletions of *N1L* and *A46R* virulence and host immune regulation genes	[64]
	*TK*	Deletion of *TK* gene to increase selective replication in cancer cells	[65]
Epstein-Barr Virus	EBNA1, OriP, W repeats	Inhibition of EBV replication and clearance of virus from infected tumour cells	[67]
	*EBNA1*, *EBNA3C*, *EBNA-LP BKRF4*	Decrease in viral load/replication	[68,69]
Human Immunodeficiency Virus-1	LTR, *Gag*, *Pol*, *Tat*, *Rev*, *Env*	Disruption of single loci partially inhibited viral replication and created escape mutants; Disruption of multiple loci completely abrogated viral replication and prevented virus escape	[70,71]
	5′-3′ LTR region	Precise removal of entire pro-viral genome spanning 5′-3′LTR from latently infected human CD4^+^ T cells; diminished viral replication in infected human primary CD4^+^ T cells	[72]
	TATA box, Transactivation Response (TAR) element, Rev Response element (RRE)	Specific changes in HIV-1 genome may avoid DSB repair of CRISPR/Cas9 introduced changes in HIV-1 and generation of resistant HIV-1 strains	[73]
	Gag, Pol, Rev, LTR	Inhibition HIV-1 infection (early phase)	[74,75]
	Gap, Pol, Env, Rev LTR, Vif,	Affects viral replication and escape	[75,76,77]
JC Polyomavirus	non-coding control region (NCCR), Capsid proteins VP1 and VP2	Editing NCCR and late region inhibits virus replication	[78]
Adenovirus (Adv-EGFP and Adv-DsRed recombinants)	Enhanced green fluorescent protein (EGFP) and Red fluorescent protein	Targeted site-specific mutations in EGFP and DsRed transgenes	[79]
Herpes Simplex Virus-1	ICP0, non-coding region UL37-UL38	ICP0 double knock out	[80]
	miRNAs –BART5, BART6 and BART16	Inhibition of HSV-1 replication	[81]
	Intergenic space UL26-UL27	Induce recombination	[82]
Human Cytomegalovirus	UL54, UL44, UL57, UL70, UL105, UL86, UL84, US6, US7, US11	Inhibition of HCMV replication	[81]
Hepatitis B Virus	Covalently closed circular DNA (cccDNA)	Inactivation of HBV cccDNA	[83,84,85,86,87]
	Several conserved genomic regions	Inhibition of viral replication	[88]
	HBV surface protein (HBsAg) encoding region	Inhibition of viral replication	[89]
	HBV core (HBcAg) and surface (HBsAg) proteins	Reduced HBV expression; clearance of virus	[90]
Human Papilloma Virus-16	E6, E7 genes, promoter of E6/E7	Reduced proliferation of HPV16-positive cells and inhibition of tumorigenicity in xenograft studies	[91]
Zika virus	24 conserved genomic regions of Zika virus	CRISPR/Cas9-based methodology to discriminate strains at single base resolution	[92]

**Table 2:** Overview of CRISPR/Cas9 applications in virus-host interaction.

Virus	Target	Effect	PMID
Hepatitis A virus	Cellular protein glucose-regulated protein 78 (GRP78)	Antiviral protein: knockout of GRP78 enhances HAV replication in Huh7 cells	[93]
Hepatitis B virus	Cellular proteins p53 and PTEN	Knockout of p53 and PTEN accelerates HBV-induced HCC in adult HBV transgenic mice	[94]
	miR-3188	KO of miR-3188 inhibited xenograft tumour growth of HBV-positive HCC in nude mice	[95]
	Complete genome	Complete removal of integrated HBV genome in HCC resulted in very low levels of supernatant HBV DNA, HBsAg and HBeAg	[96]
	CDC42	KO of CDC42 in HuH-7-HBx cells reduced proliferation mediated by pX protein of HBV	[97]
	DNA polymerase K	KO prevents conversion of relaxed circular HBV DNA into ccc DNA	[98]
	S and X genes	Reduced viral infectivity	[99,100]
	Regulatory region	Inhibits HBV replication	[101]
Hepatitis C Virus	STAT1 and STAT2 in Huh-7.5 cells	Inhibition of HCV replication by IFNλ depends on STAT1 and STAT2, while STAT2 is necessary for IFNα-induced inhibition of HCV replication	[102]
	ISG15	KO of ISG15 increases HCV replication	[103]
		CLDN1, OCLN and CD81 are necessary for cell-free entry and cell-to-cell transmission of the virus	[104]
Herpes Simplex Virus-1	LULL1	LULL1 KO reduces HSV-1genome replication 10-fold	[105]
	ICP0	Reduced viral infectivity	[106]
Human Immunodeficiency Virus-1	CCR5	KO of the CCR5 receptor in CD34+ hematopoietic stem cells makes them resistant to HIV	[107]
	Cellular genes	Interaction between capsid protein and IFNα-induced antiviral factors	[108]
	LTR and gag gene	Cleavage of integrated viral DNA resulting in eradication of the virus	[109]
	Cellular protein SAMHD1	Moe efficient HIV-1 infection in SAMHD1 KO THP-1 cells	[110]
	LTR	Remove integrated viral genome	[111,112,113]
	CCR5	KO of CCR5 co-receptor prevents HIV-1 infection	[114,115]
	LTR	Activation of latent HIV-1 infection	[116]
	CXCR4	KO of CXCR4 makes CD4^+^ cells resistant to HIV-1 infection	[117]
	ESCRT-II	KO of ESCRT-II reduces virus production and budding	[118]
	ER-Mannosidase I gene (MAN1B1)	MAN1B1 is involved in env degradation	[119]
	TSPO (mitochondrial translocator protein)	TSPO inhibits HIV-1 Env expression	[120]
Human Immunodeficiency Virus-2	BST2 (=tetherin) in H9 cells	BST2 is necessary for HIV-2 release	[121]
Epstein-Barr Virus	Multiple cellular proteins	sgRNA library was used to identify cellular targets that EBV uses to transform cells	[122]
	CD63	KO reduces exosomal package of LMP1	[123]
	BART promoter	Protocol 558 bp deletion in BART promoter	[124,125]
		Episomal EBV genome which facilitates cloning and sequencing	[126]
Ebola virus	ER chaperones calnexin and calreticulin	KO of calnexin or/and calreticulin decrease expression of EBOV glycoprotein GP in HEK293T cells	[127]
Picornavirus (polio and entero)	Multiple cellular proteins	sgRNA library was used to identify cellular targets	[128]
Zika virus	AXL (attachment factor for ZIKV)	KO of AXL has no effect on ZIKV entry	[129]
Zika and Dengue virus	ER-localized signal peptidase SEC11	Cavinafungin, an antiviral drug against Zika and Dengue viruses, inhibits signal peptidase and thereby inhibits virus replication	[130]
	Genome-wide screen of host genes	AXL, NDST1, EXT, EMC and other cellular proteins are required for viral entry	[131]
Flavivirus	Genome-wide screen of host genes	Reduce flavivirus infection: ER-associated signal peptide complex (SPCS1)	[132]
Alphaviruses	Cellular genes	Antiviral activity against alphaviruses (IFR3-STING pathway)	[133]
KSHV	Cellular protein SIRT1	KO of SIRT1 reduced cell proliferation and colony formation of KSHV-transformed cells	[134]
	Cellular Lipoxin A4 receptor (=ALX/FPR)	Effect on KSHV-mediated anti-inflammatory response	[135]
RSV	Cellular IDO (indoleamine-2,3-dioxygenase)	RSV regulates immune response of mesenchymal stem cells by upregulating expression of IDO	[136]
HCMV	Cellular protein kinase R	Viral replication	[137]
JCPyV	LTAg	Inhibition of LTAg expression inhibits viral replication	[138]
Sindbis virus	eIF2A or/and eIF2D	eIF2 KO HAF1 cells had no effect on translation of viral mRNA	[139]

## Supplementary Materials

The following are available online at http://www.mdpi.com/1999-4915/10/1/50/s1, Table S1: Examples of clinically tried orthopoxvirus-derived recombinant vaccines/vectors.
