# Supplementary material for: CRISPR/Cas9—Advancing Orthopoxvirus Genome Editing for Vaccine and Vector Development
Source: Viruses. 2018 Jan 22;10(1):50. doi: 10.3390/v10010050 (PMC5795463; doi:10.3390/v10010050)
Supplement: Supplementary file 1 [file viruses-10-00050-s001.pdf]

Supplementary Table 1: Examples of clinically tried orthopoxvirus-derived recombinant vaccines/vectors

Orthopox virus Strain	Recombinant Vector/Vaccine[1]	Intended Host	Target Disease	Description of encoded heterologous Antigen	Reference
Vaccinia	PROSTVAC-V/TRICOM; Vaccinia-B7.1	Human	Prostate; Melanoma cancer	Prostate specific antigen; and co-stimulatory T-cell molecules	[146-149]
	PANVAC-V	Human	Colorectal, breast and ovarian cancers	Transgenes for the tumor-associated antigens epithelial mucin 1 and carcinoembryonic antigen	[202]
	Raboral V-RG	Wildlife (red fox, coyote, raccoon, dog, cat)	Rabies	Rabies virus glycoprotein G	[203]
	rVac-Tyrosinase	Human	Malignant melanoma	Tyrosinase	[204]
NYVAC	NYVAC- CDV-H/F	Ferrets	Canine distemper	Canine distemper virus haemagglutinin (H) and fusion (F) protein genes	[205]
	NYVAC-EHV-1-vP1014	Horse	Equine Rhinopneumonitis	Immediate early gene (gene 64) of equine herpesvirus-1 (EHV-1)	[206]
	NYVAC-B	Human	HIV/AIDS	HIV-1 (clade B) *env*, *gag-pol-nef* and *gp120*	[207, 208]
	NYVAC-C	Human	HIV/AIDS	HIV-1 (clade C) *env*, *gag-pol-nef*	[209]
	NYVAC-Pf7	Human	Malaria	Multiple *Plasmodium falciparum* antigens, namely, *cs, ssp2, lsa1, msp1, sera, ama1, pfs25*	[210]
	NYVAC-PRV- gII/ gp50	Pig	Pseudorabies	Pseudorabies virus glycoproteins *gII* and *gp50*	[211]

	NYVAC-JEV	Human	Japanese encephalitis	Japanese encephalitis virus premembrane (*prM*), envelope (E) and non-structural 1 (NS1) proteins	[212, 213]
MVA	MVA-mBN32	Human	HIV/AIDS	21 CTL and 18 HTL epitopes from HIV-1 *gag, pol, vpr, nef, rev* and *env*	[214]
	MVA-CMDR	Human	HIV/AIDS	MVA-Chang Mai double recombinant (CMDR)<br>expressing HIV-1 *env* (clade E); *gag-pol* (clade A)	[215, 216]
	MVA.HIVA	Human	HIV/AIDS	HIV-1 (clade A) Gap *p24* and *p17* fused to 25 overlapping CTL CD8 T cell epitopes	[217]
	TBC-M4	Human	HIV/AIDS	HIV-1 (clade C) *env, gp160, gag*, RT, *rev*, *tat*, and *nef*	[218]
	ADMVA	Human	HIV/AIDS	HIV-1 (clade B/C*) env, gag-pol, nef-tat*	[219]
	GOVX-B11	Human	HIV/AIDS	HIV-1 (clade B) *gag*, PR, RT, *env*	[220]
	rMVA-HIV	Human	HIV/AIDS	HIV-1 (clade B) *env/gag; tat/rev/nef*-RT	[221]
	MVA-nef	Human	HIV/AIDS	HIV-1 *nef*	[222]
	MVA-BN®-Filo	Human	Ebola	Glycoproteins from Ebola virus, Sudan virus, Marburg virus, and nucleoprotein from Tai Forest virus	[223]
	MVA- AHS-VP2	Horse	African Horse Sickness	VP2 protein of African Horse Sickness virus	[224]
	MVA-GnGc	Lamb	Rift Valley Fever	Rift Valley Fever virus glycoproteins Gn and Gc	[225]
	MVA-brachyury- TRICOM	Human	Metastatic cancers – including lung, breast, prostate and ovarian cancers	Brachyury protein and T-cell costimulatory molecules	[226]
	MVA-NP+M1	Human	Influenza A	Influenza A nucleoprotein (NP) and matrix protein 1 (M1)	[227]

	MVA-H5-sfMR	Human	Influenza A	Haemagglutinin gene of influenza A/Vietnam/1194/2004 virus H5N1 subtype	[228]
	MVA.HBS	Human	Hepatitis B	Surface antigen of Hepatitis B virus	[229]
	MVA85A	Human	Tuberculosis	Mycobacterial mycolyl-transferase antigen 85A	[230-232]
	ChAd63-MVA-ME_TRAP/CS	Human	Malaria	*Plasmodium falciparum* pre-erythrocytic antigen ME-TRAP (multiple epitope string with thrombospondin-related adhesion protein) and CS (Circumsporozoite protein)	[233, 234]
	ChAd63-MVA AMA1	Human	Malaria	Apical membrane antigen 1 (AMA1) of *Plasmodium falciparum*	[235]
	ChAd63-MVA-MSP1/AMAI	Human	Malaria	Merozoite surface protein 1 (MSP1) and dominant over apical membrane antigen 1 (AMA1)	[236]
	MVA-PP	Human	Malaria	Polyprotein consisting of six pre-erythrocytic antigens from Plasmodium falciparum	[237]
Racoon poxvirus	RCN/rabies-G	Cat	Rabies	Rabies glycoprotein G	[238]

1 – Name given to the vector/vaccine by the developer

Sources: Clinicaltrial.gov (https://clinicaltrials.gov/ct2/search); Vaxvec: A Database of Recombinant Vaccine Vectors and Recombinant Vector Vaccines (http://www.violinet.org/vaxvec/) and PubMed

References

146. McNeel, D. G.; Chen, Y.-H.; Gulley, J. L.; Dwyer, A. J.; Madan, R. A.; Carducci, M. A.; DiPaola, R. S., Randomized phase II trial of docetaxel with or without PSA-TRICOM vaccine in patients with castrate-resistant metastatic prostate cancer: A trial of the ECOG-ACRIN cancer research group (E1809). *Human Vaccines & Immunotherapeutics* **2015,** 11, (10), 2469-2474.
147. Gulley, J. L.; Madan, R. A.; Tsang, K. Y.; Jochems, C.; Marté, J. L.; Farsaci, B.; Tucker, J. A.; Hodge, J. W.; Liewehr, D. J.; Steinberg, S. M.; Heery, C. R.; Schlom, J., Immune Impact Induced by PROSTVAC (PSA-TRICOM), a Therapeutic Vaccine for Prostate Cancer. *Cancer Immunology Research* **2014,** 2, (2), 133.

148. Arlen, P. M.; Gulley, J. L.; Parker, C.; Skarupa, L.; Pazdur, M.; Panicali, D.; Beetham, P.; Tsang, K. Y.; Grosenbach, D. W.; Feldman, J.; Steinberg, S. M.; Jones, E.; Chen, C.; Marte, J.; Schlom, J.; Dahut, W., A randomized phase II study of concurrent docetaxel plus vaccine versus vaccine alone in metastatic androgen-independent prostate cancer. *Clinical cancer research : an official journal of the American Association for Cancer Research* **2006,** 12, (4), 1260-9.
149. Kaufman, H. L.; Deraffele, G.; Mitcham, J.; Moroziewicz, D.; Cohen, S. M.; Hurst-Wicker, K. S.; Cheung, K.; Lee, D. S.; Divito, J.; Voulo, M.; Donovan, J.; Dolan, K.; Manson, K.; Panicali, D.; Wang, E.; Horig, H.; Marincola, F. M., Targeting the local tumor microenvironment with vaccinia virus expressing B7.1 for the treatment of melanoma. *The Journal of clinical investigation* **2005,** 115, (7), 1903-12.
150. Okoli, A. S., Asare, N., Gjøen, T., Klein, J., Ytrehus, B., Knowledge base for the assessment of environmental risks by the use of genetically modified virus-vectored vaccines for domesticated animals. . *Scientific Opinion of the Panel on Microbial Ecology of the Norwegian Scientific Committee for Food Safety (VKM).* **2016.**
151. Rehm, K. E.; Roper, R. L., Deletion of the A35 gene from Modified Vaccinia Virus Ankara increases immunogenicity and isotype switching. *Vaccine* **2011,** 29, (17), 3276-83.
152. Garber, D. A.; O'Mara, L. A.; Gangadhara, S.; McQuoid, M.; Zhang, X.; Zheng, R.; Gill, K.; Verma, M.; Yu, T.; Johnson, B.; Li, B.; Derdeyn, C. A.; Ibegbu, C.; Altman, J. D.; Hunter, E.; Feinberg, M. B., Deletion of Specific Immune-Modulatory Genes from Modified Vaccinia Virus Ankara-Based HIV Vaccines Engenders Improved Immunogenicity in Rhesus Macaques. *Journal of virology* **2012,** 86, (23), 12605-12615.
153. Holgado, M. P.; Falivene, J.; Maeto, C.; Amigo, M.; Pascutti, M. F.; Vecchione, M. B.; Bruttomesso, A.; Calamante, G.; Del Medico-Zajac, M. P.; Gherardi, M. M., Deletion of A44L, A46R and C12L Vaccinia Virus Genes from the MVA Genome Improved the Vector Immunogenicity by Modifying the Innate Immune Response Generating Enhanced and Optimized Specific T-Cell Responses. *Viruses* **2016,** 8, (5).
154. Staib, C.; Kisling, S.; Erfle, V.; Sutter, G., Inactivation of the viral interleukin 1beta receptor improves CD8+ T-cell memory responses elicited upon immunization with modified vaccinia virus Ankara. *The Journal of general virology* **2005,** 86, (Pt 7), 1997-2006.
155. García-Arriaza, J.; Arnáez, P.; Gómez, C. E.; Sorzano, C. Ó. S.; Esteban, M., Improving Adaptive and Memory Immune Responses of an HIV/AIDS Vaccine Candidate MVA-B by Deletion of Vaccinia Virus Genes (C6L and K7R) Blocking Interferon Signaling Pathways. *PloS one* **2013,** 8, (6), e66894.
156. Dai, P.; Wang, W.; Cao, H.; Avogadri, F.; Dai, L.; Drexler, I.; Joyce, J. A.; Li, X. D.; Chen, Z.; Merghoub, T.; Shuman, S.; Deng, L., Modified vaccinia virus Ankara triggers type I IFN production in murine conventional dendritic cells via a cGAS/STING-mediated cytosolic DNA-sensing pathway. *PLoS pathogens* **2014,** 10, (4), e1003989.
157. Zou, Z.; Huang, K.; Wei, Y.; Chen, H.; Liu, Z.; Jin, M., Construction of a highly efficient CRISPR/Cas9-mediated duck enteritis virus-based vaccine against H5N1 avian influenza virus and duck Tembusu virus infection. *Scientific reports* **2017,** 7, (1), 1478.
158. Staib, C.; Drexler, I.; Sutter, G., Construction and isolation of recombinant MVA. *Methods Mol Biol* **2004,** 269, 77-100.
159. Liang, X.; Sun, L.; Yu, T.; Pan, Y.; Wang, D.; Hu, X.; Fu, Z.; He, Q.; Cao, G., A CRISPR/Cas9 and Cre/Lox system-based express vaccine development strategy against re-emerging Pseudorabies virus. *Scientific reports* **2016,** 6, 19176.
160. Hansen, H.; Okeke, M. I.; Nilssen, O.; Traavik, T., Recombinant viruses obtained from co-infection in vitro with a live vaccinia-vectored influenza vaccine and a naturally occurring cowpox virus display different plaque phenotypes and loss of the transgene. *Vaccine* **2004,** 23, (4), 499-506.

161. Wang, Z.; Martinez, J.; Zhou, W.; La Rosa, C.; Srivastava, T.; Dasgupta, A.; Rawal, R.; Li, Z.; Britt, W. J.; Diamond, D., Modified H5 promoter improves stability of insert genes while maintaining immunogenicity during extended passage of genetically engineered MVA vaccines. *Vaccine* **2010,** 28, (6), 1547-57.
162. Antoine, G.; Scheiflinger, F.; Dorner, F.; Falkner, F. G., The complete genomic sequence of the modified vaccinia Ankara strain: comparison with other orthopoxviruses. *Virology* **1998,** 244, (2), 365-96.
163. Drexler, I.; Heller, K.; Wahren, B.; Erfle, V.; Sutter, G., Highly attenuated modified vaccinia virus Ankara replicates in baby hamster kidney cells, a potential host for virus propagation, but not in various human transformed and primary cells. *The Journal of general virology* **1998,** 79 (Pt 2), 347-52.
164. Blanchard, T. J.; Alcami, A.; Andrea, P.; Smith, G. L., Modified vaccinia virus Ankara undergoes limited replication in human cells and lacks several immunomodulatory proteins: implications for use as a human vaccine. *The Journal of general virology* **1998,** 79 (Pt 5), 1159-67.
165. Di Pilato, M.; Sanchez-Sampedro, L.; Mejias-Perez, E.; Sorzano, C. O.; Esteban, M., Modification of promoter spacer length in vaccinia virus as a strategy to control the antigen expression. *The Journal of general virology* **2015,** 96, (8), 2360-71.
166. Timm, A.; Enzinger, C.; Felder, E.; Chaplin, P., Genetic stability of recombinant MVA-BN. *Vaccine* **2006,** 24, (21), 4618-21.
167. Orubu, T.; Alharbi, N. K.; Lambe, T.; Gilbert, S. C.; Cottingham, M. G., Expression and cellular immunogenicity of a transgenic antigen driven by endogenous poxviral early promoters at their authentic loci in MVA. *PloS one* **2012,** 7, (6), e40167.
168. Gaj, T.; Gersbach, C. A.; Barbas, C. F., ZFN, TALEN, and CRISPR/Cas-based methods for genome engineering. *Trends Biotechnol* **2013,** 31.
169. Boch, J.; Bonas, U., Xanthomonas AvrBs3 family-type III effectors: discovery and function. *Annual review of phytopathology* **2010,** 48, 419-36.
170. Wayengera, M., Proviral HIV-genome-wide and pol-gene specific zinc finger nucleases: usability for targeted HIV gene therapy. *Theor Biol Med Model* **2011,** 8, 26.
171. Wayengera, M., Zinc finger arrays binding human papillomavirus types 16 and 18 genomic DNA: precursors of gene-therapeutics for in-situ reversal of associated cervical neoplasia. *Theoretical Biology and Medical Modelling* **2012,** 9, (1), 30.
172. Wayengera, M., Identity of zinc finger nucleases with specificity to herpes simplex virus type II genomic DNA: novel HSV-2 vaccine/therapy precursors. *Theor Biol Med Model* **2011,** 8, 23.
173. Bloom, K.; Ely, A.; Arbuthnot, P., A T7 Endonuclease I Assay to Detect Talen-Mediated Targeted Mutation of HBV cccDNA. *Methods Mol Biol* **2017,** 1540, 85-95.
174. Dreyer, T.; Nicholson, S.; Ely, A.; Arbuthnot, P.; Bloom, K., Improved antiviral efficacy using TALEN-mediated homology directed recombination to introduce artificial primary miRNAs into DNA of hepatitis B virus. *Biochemical and biophysical research communications* **2016,** 478, (4), 1563-8.
175. Cermak, T.; Doyle, E. L.; Christian, M.; Wang, L.; Zhang, Y.; Schmidt, C.; Baller, J. A.; Somia, N. V.; Bogdanove, A. J.; Voytas, D. F., Efficient design and assembly of custom TALEN and other TAL effector-based constructs for DNA targeting. *Nucleic Acids Res* **2011,** 39, (12), e82.
176. Morbitzer, R.; Elsaesser, J.; Hausner, J.; Lahaye, T., Assembly of custom TALE-type DNA binding domains by modular cloning. *Nucleic Acids Res* **2011,** 39, (13), 5790-9.
177. Guha, T. K.; Wai, A.; Hausner, G., Programmable Genome Editing Tools and their Regulation for Efficient Genome Engineering. *Computational and structural biotechnology journal* **2017,** 15, 146-160.

178. Guilinger, J. P.; Thompson, D. B.; Liu, D. R., Fusion of catalytically inactive Cas9 to FokI nuclease improves the specificity of genome modification. *Nat Biotechnol* **2014,** 32, (6), 577-582.
179. Jiang, W.; Bikard, D.; Cox, D.; Zhang, F.; Marraffini, L. A., RNA-guided editing of bacterial genomes using CRISPR-Cas systems. *Nat Biotechnol* **2013,** 31, (3), 233-9.
180. Mali, P.; Yang, L.; Esvelt, K. M.; Aach, J.; Guell, M.; DiCarlo, J. E.; Norville, J. E.; Church, G. M., RNA-guided human genome engineering via Cas9. *Science* **2013,** 339, (6121), 823-6.
181. Peng, R.; Lin, G.; Li, J., Potential pitfalls of CRISPR/Cas9-mediated genome editing. *FEBS J* **2016,** 283, (7), 1218-31.
182. Kim, K.; Ryu, S. M.; Kim, S. T.; Baek, G.; Kim, D.; Lim, K.; Chung, E.; Kim, S.; Kim, J. S., Highly efficient RNA-guided base editing in mouse embryos. *Nat Biotechnol* **2017,** 35, (5), 435-437.
183. Salsman, J.; Masson, J. Y.; Orthwein, A.; Dellaire, G., CRISPR/Cas9 Gene Editing: From Basic Mechanisms To Improved Strategies For Enhanced Genome Engineering In Vivo. *Current gene therapy* **2017**.
184. Song, J.; Yang, D.; Xu, J.; Zhu, T.; Chen, Y. E.; Zhang, J., RS-1 enhances CRISPR/Cas9- and TALEN-mediated knock-in efficiency. *Nature communications* **2016,** 7, 10548.
185. Tsai, S. Q.; Joung, J. K., Defining and improving the genome-wide specificities of CRISPR-Cas9 nucleases. *Nature reviews. Genetics* **2016,** 17, (5), 300-12.
186. Dang, Y.; Jia, G.; Choi, J.; Ma, H.; Anaya, E.; Ye, C.; Shankar, P.; Wu, H., Optimizing sgRNA structure to improve CRISPR-Cas9 knockout efficiency. *Genome Biology* **2015,** 16, (1), 280.
187. Fu, Y.; Foden, J. A.; Khayter, C.; Maeder, M. L.; Reyon, D.; Joung, J. K.; Sander, J. D., High-frequency off-target mutagenesis induced by CRISPR-Cas nucleases in human cells. *Nat Biotechnol* **2013,** 31, (9), 822-6.
188. Pattanayak, V.; Lin, S.; Guilinger, J. P.; Ma, E.; Doudna, J. A.; Liu, D. R., High-throughput profiling of off-target DNA cleavage reveals RNA-programmed Cas9 nuclease specificity. *Nat Biotechnol* **2013,** 31, (9), 839-43.
189. Zheng, T.; Hou, Y.; Zhang, P.; Zhang, Z.; Xu, Y.; Zhang, L.; Niu, L.; Yang, Y.; Liang, D.; Yi, F.; Peng, W.; Feng, W.; Yang, Y.; Chen, J.; Zhu, Y. Y.; Zhang, L.-H.; Du, Q., Profiling single-guide RNA specificity reveals a mismatch sensitive core sequence. *Scientific reports* **2017,** 7, 40638.
190. Wu, X.; Kriz, A. J.; Sharp, P. A., Target specificity of the CRISPR-Cas9 system. *Quantitative biology* **2014,** 2, (2), 59-70.
191. Zhang, X. H.; Tee, L. Y.; Wang, X. G.; Huang, Q. S.; Yang, S. H., Off-target Effects in CRISPR/Cas9-mediated Genome Engineering. *Molecular therapy. Nucleic acids* **2015,** 4, e264.
192. Besenbacher, S.; Liu, S.; Izarzugaza, J. M.; Grove, J.; Belling, K.; Bork-Jensen, J.; Huang, S.; Als, T. D.; Li, S.; Yadav, R.; Rubio-Garcia, A.; Lescai, F.; Demontis, D.; Rao, J.; Ye, W.; Mailund, T.; Friborg, R. M.; Pedersen, C. N.; Xu, R.; Sun, J.; Liu, H.; Wang, O.; Cheng, X.; Flores, D.; Rydza, E.; Rapacki, K.; Damm Sorensen, J.; Chmura, P.; Westergaard, D.; Dworzynski, P.; Sorensen, T. I.; Lund, O.; Hansen, T.; Xu, X.; Li, N.; Bolund, L.; Pedersen, O.; Eiberg, H.; Krogh, A.; Borglum, A. D.; Brunak, S.; Kristiansen, K.; Schierup, M. H.; Wang, J.; Gupta, R.; Villesen, P.; Rasmussen, S., Novel variation and de novo mutation rates in population-wide de novo assembled Danish trios. *Nature communications* **2015,** 6, 5969.
193. Ran, F. A.; Hsu, P. D.; Lin, C. Y.; Gootenberg, J. S.; Konermann, S.; Trevino, A. E.; Scott, D. A.; Inoue, A.; Matoba, S.; Zhang, Y.; Zhang, F., Double nicking by RNA-guided CRISPR Cas9 for enhanced genome editing specificity. *Cell* **2013,** 154, (6), 1380-9.

194. Shen, B.; Zhang, W.; Zhang, J.; Zhou, J.; Wang, J.; Chen, L.; Wang, L.; Hodgkins, A.; Iyer, V.; Huang, X.; Skarnes, W. C., Efficient genome modification by CRISPR-Cas9 nickase with minimal off-target effects. *Nat Methods* **2014,** 11, (4), 399-402.
195. Tsai, S. Q.; Wyvekens, N.; Khayter, C.; Foden, J. A.; Thapar, V.; Reyon, D.; Goodwin, M. J.; Aryee, M. J.; Joung, J. K., Dimeric CRISPR RNA-guided FokI nucleases for highly specific genome editing. *Nat Biotech* **2014,** 32, (6), 569-576.
196. Wyvekens, N.; Topkar, V. V.; Khayter, C.; Joung, J. K.; Tsai, S. Q., Dimeric CRISPR RNA-Guided FokI-dCas9 Nucleases Directed by Truncated gRNAs for Highly Specific Genome Editing. *Human gene therapy* **2015,** 26, (7), 425-31.
197. DeWitt, M. A.; Magis, W.; Bray, N. L.; Wang, T.; Berman, J. R.; Urbinati, F.; Heo, S.-J.; Mitros, T.; Muñoz, D. P.; Boffelli, D.; Kohn, D. B.; Walters, M. C.; Carroll, D.; Martin, D. I. K.; Corn, J. E., Selection-free genome editing of the sickle mutation in human adult hematopoietic stem/progenitor cells. *Science Translational Medicine* **2016,** 8, (360), 360ra134-360ra134.
198. Fu, Y.; Sander, J. D.; Reyon, D.; Cascio, V. M.; Joung, J. K., Improving CRISPR-Cas nuclease specificity using truncated guide RNAs. *Nat Biotech* **2014,** 32, (3), 279-284.
199. Haeussler, M.; Schonig, K.; Eckert, H.; Eschstruth, A.; Mianne, J.; Renaud, J. B.; Schneider-Maunoury, S.; Shkumatava, A.; Teboul, L.; Kent, J.; Joly, J. S.; Concordet, J. P., Evaluation of off-target and on-target scoring algorithms and integration into the guide RNA selection tool CRISPOR. *Genome Biol* **2016,** 17, (1), 148.
200. Liang, C.; Wainberg, M. A.; Das, A. T.; Berkhout, B., CRISPR/Cas9: a double-edged sword when used to combat HIV infection. *Retrovirology* **2016,** 13, (1), 37.
201. Zetsche, B.; Gootenberg, J. S.; Abudayyeh, O. O.; Slaymaker, I. M.; Makarova, K. S.; Essletzbichler, P.; Volz, S. E.; Joung, J.; Oost, J.; Regev, A., Cpf1 is a single RNA-guided endonuclease of a class 2 CRISPR-Cas system. *Cell* **2015,** 163.
202. Madan, R. A.; Arlen, P. M.; Gulley, J. L., PANVAC-VF: poxviral-based vaccine therapy targeting CEA and MUC1 in carcinoma. *Expert opinion on biological therapy* **2007,** 7, (4), 543-54.
203. Blancou, J.; Artois, M.; Brochier, B.; Thomas, I.; Pastoret, P. P.; Desmettre, P.; Languet, B.; Kieny, M. P., [Safety and efficacy of an antirabies vaccine consisting of recombinant vaccinia-rabies virus administered orally to the fox, dog and cat]. *Annales de recherches veterinaires. Annals of veterinary research* **1989,** 20, (2), 195-204.
204. Lindsey, K. R.; Gritz, L.; Sherry, R.; Abati, A.; Fetsch, P. A.; Goldfeder, L. C.; Gonzales, M. I.; Zinnack, K. A.; Rogers-Freezer, L.; Haworth, L.; Mavroukakis, S. A.; White, D. E.; Steinberg, S. M.; Restifo, N. P.; Panicali, D. L.; Rosenberg, S. A.; Topalian, S. L., Evaluation of prime/boost regimens using recombinant poxvirus/tyrosinase vaccines for the treatment of patients with metastatic melanoma. *Clinical cancer research : an official journal of the American Association for Cancer Research* **2006,** 12, (8), 2526-37.
205. Welter, J.; Taylor, J.; Tartaglia, J.; Paoletti, E.; Stephensen, C. B., Vaccination against canine distemper virus infection in infant ferrets with and without maternal antibody protection, using recombinant attenuated poxvirus vaccines. *Journal of virology* **2000,** 74, (14), 6358-67.
206. Paillot, R.; Ellis, S. A.; Daly, J. M.; Audonnet, J. C.; Minke, J. M.; Davis-Poynter, N.; Hannant, D.; Kydd, J. H., Characterisation of CTL and IFN-gamma synthesis in ponies following vaccination with a NYVAC-based construct coding for EHV-1 immediate early gene, followed by challenge infection. *Vaccine* **2006,** 24, (10), 1490-500.

207. Bart, P.; Huang, Y.; Frahm, N.; Karuna, S.; Allen, M.; Kochar, N.; Chappuis, S.; Gaillard, J.; Graham, B.; Pantaleo, G., rAd5 prime/NYVAC-B boost regimen is superior to NYVAC-B prime/rAd5 boost regimen for both response rates and magnitude of CD4 and CD8 T-cell responses. *Retrovirology* **2012,** 9, (2), O72.
208. Harari, A.; Rozot, V.; Cavassini, M.; Bellutti Enders, F.; Vigano, S.; Tapia, G.; Castro, E.; Burnet, S.; Lange, J.; Moog, C.; Garin, D.; Costagliola, D.; Autran, B.; Pantaleo, G.; Bart, P. A., NYVAC immunization induces polyfunctional HIV-specific T-cell responses in chronically-infected, ART-treated HIV patients. *European journal of immunology* **2012,** 42, (11), 3038-48.
209. Harari, A.; Bart, P. A.; Stohr, W.; Tapia, G.; Garcia, M.; Medjitna-Rais, E.; Burnet, S.; Cellerai, C.; Erlwein, O.; Barber, T.; Moog, C.; Liljestrom, P.; Wagner, R.; Wolf, H.; Kraehenbuhl, J. P.; Esteban, M.; Heeney, J.; Frachette, M. J.; Tartaglia, J.; McCormack, S.; Babiker, A.; Weber, J.; Pantaleo, G., An HIV-1 clade C DNA prime, NYVAC boost vaccine regimen induces reliable, polyfunctional, and long-lasting T cell responses. *The Journal of experimental medicine* **2008,** 205, (1), 63-77.
210. Ockenhouse, C. F.; Sun, P. F.; Lanar, D. E.; Wellde, B. T.; Hall, B. T.; Kester, K.; Stoute, J. A.; Magill, A.; Krzych, U.; Farley, L.; Wirtz, R. A.; Sadoff, J. C.; Kaslow, D. C.; Kumar, S.; Church, L. W.; Crutcher, J. M.; Wizel, B.; Hoffman, S.; Lalvani, A.; Hill, A. V.; Tine, J. A.; Guito, K. P.; de Taisne, C.; Anders, R.; Ballou, W. R.; et al., Phase I/IIa safety, immunogenicity, and efficacy trial of NYVAC-Pf7, a pox-vectored, multiantigen, multistage vaccine candidate for Plasmodium falciparum malaria. *The Journal of infectious diseases* **1998,** 177, (6), 1664-73.
211. Brockmeier, S. L.; Mengeling, W. L., Comparison of the protective response induced by NYVAC vaccinia recombinants expressing either gp50 or gII and gp50 of pseudorabies virus. *Canadian journal of veterinary research = Revue canadienne de recherche veterinaire* **1996,** 60, (4), 315-7.
212. Konishi, E.; Kurane, I.; Mason, P. W.; Shope, R. E.; Kanesa-Thasan, N.; Smucny, J. J.; Hoke, C. H., Jr.; Ennis, F. A., Induction of Japanese encephalitis virus-specific cytotoxic T lymphocytes in humans by poxvirus-based JE vaccine candidates. *Vaccine* **1998,** 16, (8), 842-9.
213. Kanesa-thasan, N.; Smucny, J. J.; Hoke, C. H.; Marks, D. H.; Konishi, E.; Kurane, I.; Tang, D. B.; Vaughn, D. W.; Mason, P. W.; Shope, R. E., Safety and immunogenicity of NYVAC-JEV and ALVAC-JEV attenuated recombinant Japanese encephalitis virus--poxvirus vaccines in vaccinia-nonimmune and vaccinia-immune humans. *Vaccine* **2000,** 19, (4-5), 483-91.
214. Gorse, G. J.; Newman, M. J.; deCamp, A.; Hay, C. M.; De Rosa, S. C.; Noonan, E.; Livingston, B. D.; Fuchs, J. D.; Kalams, S. A.; Cassis-Ghavami, F. L.; the, N. H. I. V. V. T. N., DNA and Modified Vaccinia Virus Ankara Vaccines Encoding Multiple Cytotoxic and Helper T-Lymphocyte Epitopes of Human Immunodeficiency Virus Type 1 (HIV-1) Are Safe but Weakly Immunogenic in HIV-1-Uninfected, Vaccinia Virus-Naive Adults. *Clinical and Vaccine Immunology : CVI* **2012,** 19, (5), 649-658.
215. Nilsson, C.; Godoy-Ramirez, K.; Hejdeman, B.; Bråve, A.; Gudmundsdotter, L.; Hallengärd, D.; Currier, J. R.; Wieczorek, L.; Hasselrot, K.; Earl, P. L.; Polonis, V. R.; Marovich, M. A.; Robb, M. L.; Sandström, E.; Wahren, B.; Biberfeld, G., Broad and Potent Cellular and Humoral Immune Responses After a Second Late HIV-Modified Vaccinia Virus Ankara Vaccination in HIV-DNA-Primed and HIV-Modified Vaccinia Virus Ankara-Boosted Swedish Vaccinees. *AIDS Research and Human Retroviruses* **2014,** 30, (3), 299-311.
216. Currier, J. R.; Ngauy, V.; de Souza, M. S.; Ratto-Kim, S.; Cox, J. H.; Polonis, V. R.; Earl, P.; Moss, B.; Peel, S.; Slike, B.; Sriplienchan, S.; Thongcharoen, P.; Paris, R. M.; Robb, M. L.; Kim, J.; Michael, N. L.; Marovich, M. A., Phase I Safety and Immunogenicity Evaluation of MVA-CMDR, a Multigenic, Recombinant Modified Vaccinia Ankara-HIV-1 Vaccine Candidate. *PloS one* **2010,** 5, (11), e13983.

217. Afolabi, M. O.; Ndure, J.; Drammeh, A.; Darboe, F.; Mehedi, S.-R.; Rowland-Jones, S. L.; Borthwick, N.; Black, A.; Ambler, G.; John-Stewart, G. C.; Reilly, M.; Hanke, T.; Flanagan, K. L., A Phase I Randomized Clinical Trial of Candidate Human Immunodeficiency Virus type 1 Vaccine MVA.HIVA Administered to Gambian Infants. *PloS one* **2013,** 8, (10), e78289.
218. Mehendale, S.; Thakar, M.; Sahay, S.; Kumar, M.; Shete, A.; Sathyamurthi, P.; Verma, A.; Kurle, S.; Shrotri, A.; Gilmour, J.; Goyal, R.; Dally, L.; Sayeed, E.; Zachariah, D.; Ackland, J.; Kochhar, S.; Cox, J. H.; Excler, J. L.; Kumaraswami, V.; Paranjape, R.; Ramanathan, V. D., Safety and immunogenicity of DNA and MVA HIV-1 subtype C vaccine prime-boost regimens: a phase I randomised Trial in HIV-uninfected Indian volunteers. *PloS one* **2013,** 8, (2), e55831.
219. Vasan, S.; Schlesinger, S. J.; Chen, Z.; Hurley, A.; Lombardo, A.; Than, S.; Adesanya, P.; Bunce, C.; Boaz, M.; Boyle, R.; Sayeed, E.; Clark, L.; Dugin, D.; Boente-Carrera, M.; Schmidt, C.; Fang, Q.; LeiBa; Huang, Y.; Zaharatos, G. J.; Gardiner, D. F.; Caskey, M.; Seamons, L.; Ho, M.; Dally, L.; Smith, C.; Cox, J.; Gill, D.; Gilmour, J.; Keefer, M. C.; Fast, P.; Ho, D. D., Phase 1 safety and immunogenicity evaluation of ADMVA, a multigenic, modified vaccinia Ankara-HIV-1 B'/C candidate vaccine. *PloS one* **2010,** 5, (1), e8816.
220. Goepfert, P. A.; Elizaga, M. L.; Seaton, K.; Tomaras, G. D.; Montefiori, D. C.; Sato, A.; Hural, J.; DeRosa, S. C.; Kalams, S. A.; McElrath, M. J.; Keefer, M. C.; Baden, L. R.; Lama, J. R.; Sanchez, J.; Mulligan, M. J.; Buchbinder, S. P.; Hammer, S. M.; Koblin, B. A.; Pensiero, M.; Butler, C.; Moss, B.; Robinson, H. L.; the, H. S. G.; the National Institutes of, A.; Infectious Diseases, H. I. V. V. T. N.; Donastorg, Y.; Qin, L.; Lawrence, D.; Cardinali, M.; Bae, J.; Holt, R.; Redinger, H.; Johannessen, J.; Broder, G.; Moody-White, J.; McKay, B.; Calazans, G.; Bentley, C.; Kakinami, L.; Skibinski, K.; Estep, S.; Tseng, J.; Swenson, M.; Madenwald, T.; Overton, E. T.; Edupuganti, S.; Rouphael, N.; Whitaker, J.; Hay, C. M.; Bunce, C. A.; Gonzales, P.; Hurtado, J. C.; Dolin, R.; Mayer, K.; Walsh, S.; Johnson, J., Specificity and 6-Month Durability of Immune Responses Induced by DNA and Recombinant Modified Vaccinia Ankara Vaccines Expressing HIV-1 Virus-Like Particles. *The Journal of infectious diseases* **2014,** 210, (1), 99-110.
221. Keefer, M. C.; Frey, S. E.; Elizaga, M.; Metch, B.; De Rosa, S. C.; Barroso, P. F.; Tomaras, G.; Cardinali, M.; Goepfert, P.; Kalichman, A.; Philippon, V.; McElrath, M. J.; Jin, X.; Ferrari, G.; Defawe, O. D.; Mazzara, G. P.; Montefiori, D.; Pensiero, M.; Panicali, D. L.; Corey, L., A phase I trial of preventive HIV vaccination with heterologous poxviral-vectors containing matching HIV-1 inserts in healthy HIV-uninfected subjects. *Vaccine* **2011,** 29, (10), 1948-58.
222. Kutscher, S.; Allgayer, S.; Dembek, C. J.; Bogner, J. R.; Protzer, U.; Goebel, F. D.; Erfle, V.; Cosma, A., MVA-nef induces HIV-1-specific polyfunctional and proliferative T-cell responses revealed by the combination of short- and long-term immune assays. *Gene Ther* **2010,** 17, (11), 1372-1383.
223. Milligan, I. D.; Gibani, M. M.; Sewell, R.; Clutterbuck, E. A.; Campbell, D.; Plested, E.; Nuthall, E.; Voysey, M.; Silva-Reyes, L.; McElrath, M. J.; De Rosa, S. C.; Frahm, N.; Cohen, K. W.; Shukarev, G.; Orzabal, N.; van Duijnhoven, W.; Truyers, C.; Bachmayer, N.; Splinter, D.; Samy, N.; Pau, M. G.; Schuitemaker, H.; Luhn, K.; Callendret, B.; Van Hoof, J.; Douoguih, M.; Ewer, K.; Angus, B.; Pollard, A. J.; Snape, M. D., Safety and Immunogenicity of Novel Adenovirus Type 26- and Modified Vaccinia Ankara-Vectored Ebola Vaccines: A Randomized Clinical Trial. *Jama* **2016,** 315, (15), 1610-23.
224. Castillo-Olivares, J.; Calvo-Pinilla, E.; Casanova, I.; Bachanek-Bankowska, K.; Chiam, R.; Maan, S.; Nieto, J. M.; Ortego, J.; Mertens, P. P., A modified vaccinia Ankara virus (MVA) vaccine expressing African horse sickness virus (AHSV) VP2 protects against AHSV challenge in an IFNAR -/- mouse model. *PloS one* **2011,** 6, (1), e16503.
225. Busquets, N.; Lorenzo, G.; Lopez-Gil, E.; Rivas, R.; Solanes, D.; Galindo-Cardiel, I.; Abad, F. X.; Rodriguez, F.; Bensaid, A.; Warimwe, G.; Gilbert, S. C.; Domingo, M.; Brun, A., Efficacy assessment of an MVA vectored Rift Valley Fever vaccine in lambs. *Antiviral research* **2014,** 108, 165-72.

226. Fernando, R. I.; Litzinger, M.; Trono, P.; Hamilton, D. H.; Schlom, J.; Palena, C., The T-box transcription factor Brachyury promotes epithelial-mesenchymal transition in human tumor cells. *The Journal of clinical investigation* **2010,** 120, (2), 533-44.
227. Antrobus, R. D.; Berthoud, T. K.; Mullarkey, C. E.; Hoschler, K.; Coughlan, L.; Zambon, M.; Hill, A. V. S.; Gilbert, S. C., Coadministration of Seasonal Influenza Vaccine and MVA-NP+M1 Simultaneously Achieves Potent Humoral and Cell-Mediated Responses. *Molecular Therapy* **2014,** 22, (1), 233-238.
228. Kreijtz, J. H.; Goeijenbier, M.; Moesker, F. M.; van den Dries, L.; Goeijenbier, S.; De Gruyter, H. L.; Lehmann, M. H.; Mutsert, G.; van de Vijver, D. A.; Volz, A.; Fouchier, R. A.; van Gorp, E. C.; Rimmelzwaan, G. F.; Sutter, G.; Osterhaus, A. D., Safety and immunogenicity of a modified-vaccinia-virus-Ankara-based influenza A H5N1 vaccine: a randomised, double-blind phase 1/2a clinical trial. *The Lancet. Infectious diseases* **2014,** 14, (12), 1196-207.
229. Cavenaugh, J. S.; Awi, D.; Mendy, M.; Hill, A. V. S.; Whittle, H.; McConkey, S. J., Partially Randomized, Non-Blinded Trial of DNA and MVA Therapeutic Vaccines Based on Hepatitis B Virus Surface Protein for Chronic HBV Infection. *PloS one* **2011,** 6, (2), e14626.
230. Meyer, J.; Harris, S. A.; Satti, I.; Poulton, I. D.; Poyntz, H. C.; Tanner, R.; Rowland, R.; Griffiths, K. L.; Fletcher, H. A.; McShane, H., Comparing the safety and immunogenicity of a candidate TB vaccine MVA85A administered by intramuscular and intradermal delivery. *Vaccine* **2013,** 31, (7), 1026-33.
231. Tameris, M.; Geldenhuys, H.; Luabeya, A. K.; Smit, E.; Hughes, J. E.; Vermaak, S.; Hanekom, W. A.; Hatherill, M.; Mahomed, H.; McShane, H.; Scriba, T. J., The candidate TB vaccine, MVA85A, induces highly durable Th1 responses. *PloS one* **2014,** 9, (2), e87340.
232. Tameris, M. D.; Hatherill, M.; Landry, B. S.; Scriba, T. J.; Snowden, M. A.; Lockhart, S.; Shea, J. E.; McClain, J. B.; Hussey, G. D.; Hanekom, W. A.; Mahomed, H.; McShane, H., Safety and efficacy of MVA85A, a new tuberculosis vaccine, in infants previously vaccinated with BCG: a randomised, placebo-controlled phase 2b trial. *Lancet (London, England)* **2013,** 381, (9871), 1021-8.
233. Ogwang, C.; Afolabi, M.; Kimani, D.; Jagne, Y. J.; Sheehy, S. H.; Bliss, C. M.; Duncan, C. J.; Collins, K. A.; Garcia Knight, M. A.; Kimani, E.; Anagnostou, N. A.; Berrie, E.; Moyle, S.; Gilbert, S. C.; Spencer, A. J.; Soipei, P.; Mueller, J.; Okebe, J.; Colloca, S.; Cortese, R.; Viebig, N. K.; Roberts, R.; Gantlett, K.; Lawrie, A. M.; Nicosia, A.; Imoukhuede, E. B.; Bejon, P.; Urban, B. C.; Flanagan, K. L.; Ewer, K. J.; Chilengi, R.; Hill, A. V.; Bojang, K., Safety and immunogenicity of heterologous prime-boost immunisation with Plasmodium falciparum malaria candidate vaccines, ChAd63 ME-TRAP and MVA ME-TRAP, in healthy Gambian and Kenyan adults. *PloS one* **2013,** 8, (3), e57726.
234. Hodgson, S. H.; Ewer, K. J.; Bliss, C. M.; Edwards, N. J.; Rampling, T.; Anagnostou, N. A.; de Barra, E.; Havelock, T.; Bowyer, G.; Poulton, I. D.; de Cassan, S.; Longley, R.; Illingworth, J. J.; Douglas, A. D.; Mange, P. B.; Collins, K. A.; Roberts, R.; Gerry, S.; Berrie, E.; Moyle, S.; Colloca, S.; Cortese, R.; Sinden, R. E.; Gilbert, S. C.; Bejon, P.; Lawrie, A. M.; Nicosia, A.; Faust, S. N.; Hill, A. V. S., Evaluation of the Efficacy of ChAd63-MVA Vectored Vaccines Expressing Circumsporozoite Protein and ME-TRAP Against Controlled Human Malaria Infection in Malaria-Naive Individuals. *The Journal of infectious diseases* **2015,** 211, (7), 1076-1086.
235. Sheehy, S. H.; Duncan, C. J.; Elias, S. C.; Biswas, S.; Collins, K. A.; O'Hara, G. A.; Halstead, F. D.; Ewer, K. J.; Mahungu, T.; Spencer, A. J.; Miura, K.; Poulton, I. D.; Dicks, M. D.; Edwards, N. J.; Berrie, E.; Moyle, S.; Colloca, S.; Cortese, R.; Gantlett, K.; Long, C. A.; Lawrie, A. M.; Gilbert, S. C.; Doherty, T.; Nicosia, A.; Hill, A. V.; Draper, S. J., Phase Ia clinical evaluation of the safety and immunogenicity of the Plasmodium falciparum blood-stage antigen AMA1 in ChAd63 and MVA vaccine vectors. *PloS one* **2012,** 7, (2), e31208.
236. Sheehy, S. H.; Duncan, C. J.; Elias, S. C.; Choudhary, P.; Biswas, S.; Halstead, F. D.; Collins, K. A.; Edwards, N. J.; Douglas, A. D.; Anagnostou, N. A.; Ewer, K. J.; Havelock, T.; Mahungu, T.; Bliss, C. M.; Miura, K.; Poulton, I. D.; Lillie, P. J.; Antrobus, R. D.; Berrie, E.; Moyle, S.; Gantlett, K.; Colloca, S.;

Cortese, R.; Long, C. A.; Sinden, R. E.; Gilbert, S. C.; Lawrie, A. M.; Doherty, T.; Faust, S. N.; Nicosia, A.; Hill, A. V.; Draper, S. J., ChAd63-MVA-vectored blood-stage malaria vaccines targeting MSP1 and AMA1: assessment of efficacy against mosquito bite challenge in humans. *Molecular therapy : the journal of the American Society of Gene Therapy* **2012,** 20, (12), 2355-68.

237. Porter, D. W.; Thompson, F. M.; Berthoud, T. K.; Hutchings, C. L.; Andrews, L.; Biswas, S.; Poulton, I.; Prieur, E.; Correa, S.; Rowland, R.; Lang, T.; Williams, J.; Gilbert, S. C.; Sinden, R. E.; Todryk, S.; Hill, A. V., A human Phase I/IIa malaria challenge trial of a polyprotein malaria vaccine. *Vaccine* **2011,** 29, (43), 7514-22.
238. Osorio, J. E.; Frank, R. S.; Moss, K.; Taraska, T.; Powell, T.; Stinchcomb, D. T., Raccoon poxvirus as a mucosal vaccine vector for domestic cats. *J Drug Target* **2003,** 11, (8-10), 463-70.

© 2018 by the authors. Licensee MDPI, Basel, Switzerland. This article is an open access article distributed under the terms and conditions of the Creative Commons Attribution (CC BY) license (http://creativecommons.org/licenses/by/4.0/).
